# The Role of Curcumin in Prevention and Management of Metastatic Disease

**DOI:** 10.3390/ijms19061716

**Published:** 2018-06-09

**Authors:** Beatrice E. Bachmeier, Peter H. Killian, Dieter Melchart

**Affiliations:** 1 Competence Center for Complementary Medicine and Naturopathy (CoCoNat), the Technical University, 80801 Munich, Germany; dieter.melchart@tum.de; 2 Institute of Laboratory Medicine, Ludwig-Maximilians-University, 81377 Munich, Germany; peterhanskillian@gmail.com; 3 Institute for Complementary and Integrative Medicine, University Hospital Zurich and University of Zurich, Zurich CH-8091, Switzerland

**Keywords:** curcumin, cancer, metastases, prevention

## Abstract

In the last two decades, targeted therapies have enhanced tumor patient care and treatment success, however, metastatic growth still cannot be stopped efficiently and, therefore, mortality rates remain high. Prevention strategies against formation of metastases are the most promising approach we have, however, due to lack of clinical validation studies, they have not yet entered routine clinical care. In order to smooth the way for efficient prevention, further preclinical and large clinical studies are required. In this context, the underlying molecular mechanisms and factors that lead to metastatic growth have to be explored, and potential preventive agents have to be tested. Thereby, special attention has to be paid to natural bioactive compounds which do not exert major adverse effects, like the plant-derived polyphenol Curcumin, which is known to be a powerful antitumor agent. So far, most of the preclinical studies with Curcumin have focused on its effect on inhibiting tumor cell proliferation and invasion, although, it is known that it also inhibits metastatic spread in vivo. This review discusses the preventive potential of this natural compound not only against tumor onset, but also against formation of metastases.

## 1. Introduction

The number of tumor cases are continuously rising, and while today there are 14.1 million new cancer cases registered worldwide, it is expected to be 23.6 million in the year 2030 (International Agency for Research on Cancer: http://globocan.iarc.fr/Pages/fact_sheets_cancer.aspx; accessed on May 2018). At present, 8.2 million persons die due to metastases from malignant tumors. In most cases, formation of metastases is an irreversible process accompanied by therapy resistance [1].

At present, tumors in the metastatic setting are treated predominantly with chemotherapy, which exerts harmful side effects and, in many cases, results in therapy resistance, confronting the vast majority of patients with a terminal illness that is to a large extent incurable by current therapeutic regimens.

Therefore, it is more beneficial to prevent cancer and its progression to the metastatic disease before this process starts. Efficient prevention strategies comprise the use of pharmacological agents or bioactive substances to impede, arrest, or reverse tumorigenesis at its early stage, which is the definition for chemoprevention, a term introduced in the early 1990s by Michael Sporn [2]. Since these days, chemoprevention was successful in many preclinical and clinical studies [3]. The outcome was that chemopreventive agents—which are natural substances, molecules, or synthetic derivates with negligible side effects—can control signal transduction and protein expression and thereby exert antitumor and antimetastatic potency.

While conventional cancer therapies in terms of synthesized antineoplastic drugs, which can be targeted (e.g., antibodies) or nontargeted (e.g., most chemotherapeutic agents), are accompanied by severe side effects and drug resistance, prevention is a strategy that applies predominantly plant-derived nontoxic natural substances or synthetic molecules with preclinically proven antitumorigenic effects and low side effects.

The term “prevention” was originally used in health management and was then translated into cancer patients’ care. Prevention is divided into primary, secondary, and tertiary prevention. The definitions are:

Primary prevention aims to prevent disease or injury before it ever occurs. It refers to activities or measures that are directed at reducing the risk of exposure to a risk factor or health determinant.

Secondary prevention aims to reduce the impact of a disease or injury that has already occurred. This is done by detecting and treating disease or injury as soon as possible to halt or slow its progress. In terms of cancer this would apply at the stage of tumor initiation in order to prevent progression.

Tertiary prevention aims to soften the impact of an ongoing illness or injury that has lasting effects. This is done by helping people manage long-term, often-complex health problems in order to improve their ability to function, their quality of life and their life expectancy (https://www.iwh.on.ca/wrmb/primary-secondary-and-tertiary-prevention, accessed on 8 June 2018).

Translated into terms of cancer patients’ care, primary prevention encompasses all interventions that impede the development of the cancerous process and includes, for example, lifestyle counseling and coaching and product safety. All sets of interventions leading to the discovery and control of cancerous or precancerous processes, like screening, early detection, and effective treatment, belong to secondary prevention [4]. The goal of tertiary prevention is to reduce morbidity and disability in people diagnosed with, and being treated for, cancer. An additional challenge of tertiary prevention is to increase the overall efficiency of patient treatment through the inclusion of interventions in order to prevent comorbidities, risk of recurrence, and second cancers.

The polyphenol Curcumin (diferuloylmethane) is extracted from the plant turmeric (*Curcuma longa*) and widely used as a spice component. The antioxidant compound is applied in traditional Indian and Chinese medicine to treat inflammatory disorders [5]. Current preclinical and clinical studies revealed that Curcumin exerts antiproliferative and proapoptotic effects against various tumors in vitro [6,7,8] and in vivo, and that it suppresses carcinogenesis of the breast [9] and other organs [10,11,12]. Curcumin is able to suppress cancer cell growth by interfering with the tumor cell cycle [13] and inhibits tumor cell invasion through regulation of cytokines, growth factors and their receptors, enzymes, and adhesion molecules [14,15,16,17,18,19]. Furthermore, Curcumin modulates the activity of transcription factors and their signaling pathways, as well as oncogenes and tumor suppressor genes [18,20]. Our group has extensively studied the preventive effect of Curcumin on metastatic growth in various in vivo models of breast and prostate cancer [14,16] and the underlying molecular mechanisms [14,15,21]. Because of its potency against tumor progression and formation of metastases and its negligible side effects, Curcumin is highly suitable for chemoprevention of cancer on all levels embracing primary, secondary, and tertiary prevention.

By preventing or retarding tumor initiation, progression, metastatic disease, and comorbidities, people can have high quality of life instead of waiting until the malignancy proceeds and only antineoplastic therapies with huge side effects have to be applied. The goal of cancer prevention is to expand the lifespan with high quality of life, where people might then die of another disease of advanced age.

## 2. Chemopreventive Properties of Curcumin

Many preclinical studies in the last 20 years have focused on unraveling the molecular mechanisms of the antitumorigenic properties of Curcumin in order to evaluate the chemopreventive potential of this natural bioactive compound [14,15,16,21,22,23,24].

The chemopreventive activities of Curcumin involve a variety of mechanisms playing a role in tumorigenesis and tumor progres­sion, for example, strong antioxidant effect by direct scavenge of reactive oxygen species, upregulation of carcinogen-detoxifying enzymes/antioxidants, as well as activation of programmed cell death (apoptosis), and the inhibition of various transcription factors with nuclear factor-κB (NFκB). It has been shown by us and others that Curcumin is able to modulate several signal transcription pathways and mainly acts by inhibiting the activity of NFκB, a transcription factor associated with inflammatory diseases and tumor progression [13,14,15,16,24].

In particular, the inhibition of the NFκB pathway by Curcumin leads to the alteration of various tumor-associated genes, gene products, and noncoding RNAs (miRNAs). In this context, proinflammatory cytokines and the mRNA expression of pro-metastatic enzymes, such as matrix metalloproteinases (MMPs) are inhibited. Similarly, other factors/enzymes, such as cyclooxygenase (COX)-2, are repressed and several other important cellular effectors are influenced. In consequence, Curcumin has been shown to interfere with a multitude of tumor-associated processes, including inflammation, cell cycle, apoptosis, cell survival, proliferation (by growth factor receptor inhibition), invasion, and metastasis [15,16].

**Figure 1:**
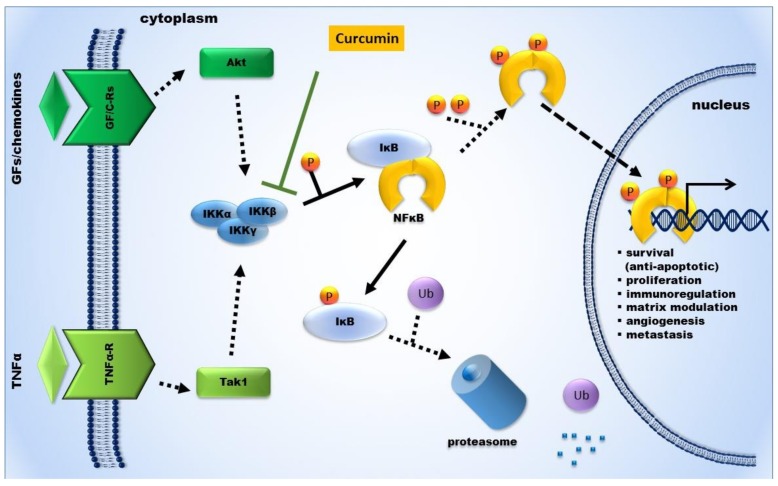
Schematic illustration of nuclear factor-κB (NFκB) signaling in the tumor environment. In resting cells, the NFκB dimer is bound to its inhibitor (IκB) and remains inactive in the cytoplasm. The activation cascade starts through binding of a ligand (TNFα = tumor necrosis factor α, TNFαR = TNFα-receptor, GF/CR = growth-factor/chemokine-receptor) to its receptor. Activation via TNFα occurs mainly through Tak1 (TGFβ-activated kinase 1) and via growth factors and/or cytokines, mainly through Akt (v-akt murine thymoma viral oncogene homolog). This leads to activation of the IKK complex (inhibitor of κB kinases; IKKα, β, γ). By activating the IKK-complex, the inhibitor of κB (IκB) is phosphorylated and the NFκB dimer is released and phosphorylated, which leads to translocation into the nucleus, where NFκB binds to the respective DNA promotor regions, which leads to transcription of genes related to proliferation, immunoregulation, matrix modulation, angiogenesis, and formation of metastasis. Curcumin inhibits the activation of NFκB by blocking the IKK-complex.

**Figure 2:**
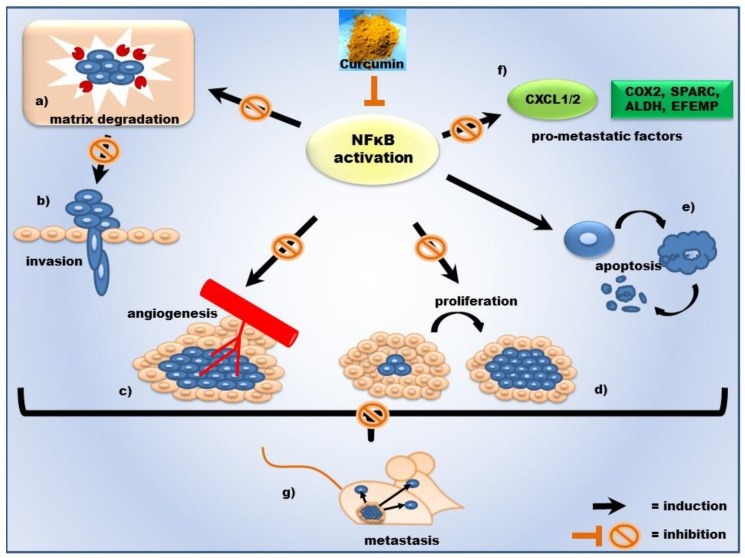
Schematic illustration of the effects of curcumin on prostate cancer. Curcumin has various effects on the development and progression of cancer via inhibition of NFκB signaling, followed by a downregulation of its target genes. Curcumin reduces tumor cell proliferation, induces apoptosis (**d**,**e**), and inhibits angiogenesis (**c**). Additionally, Curcumin inhibits gene expression of matrix-degrading proteases (matrix metalloproteases) and thereby invasion of tumor cells (**a**,**b**). Furthermore, the expression of metastasis-associated genes is reduced (**f**). As a summation of the single effects, the inhibition of lung metastasis in an animal model (murine mouse model) can be considered (**g**).

We have extensively investigated the NFκB pathway preclinically in cell models of metastatic breast and prostate cancer. Curcumin prevents NFκB activation by blocking phosphorylation and degradation of IκBα, the inhibitor of κBα. Thereby, the NFκB subunit p65 does not get phosphorylated and does not translocate into the nucleus [14,15]. In consequence, tumor-associated genes and gene products are not expressed (Figure 1). NFκB-regulated gene products that control tumor cell invasion are, among others, matrix-degrading proteases like matrix metalloproteinases (MMPs) and urokinase-type plasminogen activator (uPA) or inflammatory cytokines [25]. The inhibitory effect of Curcumin on NFκB leads to reduced tumor cell proliferation and growth and induced apoptosis. The concerted action of all these effects prevents tumor cell invasion and metastases, as illustrated in Figure 2, based on our results on metastatic prostate cancer cells [14]. The inhibition of the transcription factor NFκB is therefore a major target for prevention of cancer onset and progression.

### 2.1. Curcumin Induces Apoptosis and Inhibits Proliferation and Cell Growth

The loss of a fully functional apoptotic program of malignant tumor cells is a major target of chemoprevention of cancer. Apoptosis can be initiated by p53 (intrinsic pathway), as well as through a variety of extracellular signals (extrinsic pathway). The caspase-3, -8 and -9 are activated upon an interplay between the extrinsic and the intrinsic apoptotic pathways. In addition, the signaling pathways of PI3K/Akt (Phosphatidylinositol-3-Kinase and Protein Kinase B), MAPK (Mitogen-Activated Protein Kinase), and NFκB, which control cell proliferation and survival, are indirectly involved in the regulation of apoptotic cell death.

Curcumin has been found to suppress carcinogenesis of the breast [26] and other organs [10,11,12] in vivo and against diverse tumors in vitro [6,7,8,27], exerting significant antiproliferative and proapoptotic effects. Curcumin-induced activation of caspases and release of cytochrome C [28] and the repression of cell survival factors via the inhibition of the NFκB pathway (see below) are discussed as molecular mechanisms underlying the strong anticancer effect of Curcumin. The plant-derived polyphenol activated caspases-3, -7, -8, and -9 in several colon cancer cell lines, but reduced activation of caspases related to the mitochondrial pathway, together with a partial blocking of apoptosis-inducing factor (AIF) [29]. Curcumin has been shown to increase the permeability of the mitochondrial membrane and the collapse of its membrane potential. Further effects of Curcumin include the inhibition of Akt/protein kinase B (PKB) phosphorylation in breast cancer cells, leading to increased apoptosis [30].

Downregulation of cyclin A, G2/M cell cycle arrest, and upregulation of the CDK inhibitor p21 and Cdc2 have been observed in human colon and bladder cells [31,32]. Moreover, Curcumin treatment suppressed Bcl-X_L_, cyclin, and cyclin-dependent kinase (CDK)1 levels, increased cleavage of PARP (Poly ADP (Adenosine Diphosphate)-Ribose Polymerase), and reduced mitochondrial membrane potential [33,34]. In a preclinical study with androgen-sensitive LnCaP and androgen-insensitive PC3 prostate carcinoma cells, Curcumin induced cell cycle arrest followed by the induction of apoptosis by cyclin-dependent kinase inhibitor (CIP) p21^(WAF1/CIP1)^ (WAF1: wild-type p53-activated factor 1) [35].

Curcumin and tumor necrosis factor-related apoptosis-inducing ligand (TRAIL)/Apo2L interact to induce cytotoxicity in the prostate cancer cell line LnCaP [36]. It has been shown in an animal model that the polyphenol sensitizes even TRAIL-resistant prostate cancer cells to undergo apoptosis. The respective mechanistic in vitro studies revealed that Curcumin induces expression of TRAIL-R2/DR5, TRAIL-R1/DR4, Bax, Bak, p21^WAF1^, and p27^KIP1^( Cyclin-dependent kinase inhibitor 1B), and inhibits synthesis of NFκB and its gene products, such as Bcl-2 and Bcl-X_L_, cyclin D1, vascular endothelial growth factor (VEGF), uPA, MMP-2, MMP-9 [37,38], whereby Bax and Bak seem to be essential for maximum apoptotic response to Curcumin [39].

The involvement of Curcumin in inhibiting expression of phosphatidylinositol-3 kinase (PI3K) p110 and p85 subunits, and phosphorylation of Ser 473 Akt/PKB, has been demonstrated by Shanker and colleagues. In this study, they show that Curcumin upregulates p53 expression and its phosphorylation at serine 15 in prostate cancer cells. Through the concerted action on all these factors and the mitochondrial death pathway, Curcumin induces apoptosis [40].

### 2.2. Curcumin Inhibits MMP Expression and Thereby Tumor Cell Invasion

Proteolytic enzymes such as matrix metalloproteinases (MMPs) are capable of degrading basal membrane (BM) and extra cellular matrix (ECM) components and are therefore responsible for the destruction of peritumoral matrix enabling the invasive growth pattern [41]. Thereby, MMPs facilitate invasion and metastases by promoting migration of tumor cells from the primary tumor site into the surrounding tissue and secondary distant organ sites by entering the blood stream and the lymphatic system.

We and others have shown that the expression of MMPs correlates with the growth behavior of tumor cells in vitro and in vivo [42,43,44,45]. MMPs are involved in many biological processes covering many aspects of tumor progression, such as growth factor activation [46], tumor growth, invasion, tumor-associated inflammation, angiogenesis, and metastasis [47,48]. The control of MMP expression and activation is therefore an important target for cancer prevention.

Inhibition of MMP expression starts at the level of gene transcription. The promoter regions of the genes en­coding for MMP-1, -2, -3, -7, -9, -12, and -13 contain a proximal activating-protein-1 (AP-1) binding site approximately 70 bp 5′ to the transcription start [49,50], and most of them are NFκB-like elements [51,52].

The effect of Curcumin on MMP expression and activity has been studied extensively in vitro and in vivo in inflammatory diseases [53,54] and a series of cancer cell lines [16,55,56,57,58,59]. Curcumin suppresses synthesis of many MMPs through downregulation of AP-1 and NFκB [16]. Kim and coworkers demonstrated that Curcumin inhibits 12-*O*-tetradecanoylphorbol-13-acetate (TPA)-induced MMP-9 expression and cell invasion by suppressing NFκB and AP-1 activation [60].

Our own studies revealed that Curcumin treatment of breast cancer cells resulted in strongly diminished levels of MMP-1 and MMP-2 mRNA and protein, while MMP-3 and -9 expression levels have not been reduced considerably [16]. Similarly, proteolytic activities of the gelatinases MMP-2 and -9, as evidenced as gelatinolytic activity by zymography, were downregulated upon Curcumin treatment. Another group showed that all three curcuminoids (demethoxycurcumin, bisdemethoxycurcumin, and curcumin) significantly inhibited the expressions of MMP-2 and MMP-9, but not urokinase plasmin activator (uPA) activity and in vitro invasiveness of human fibrosarcoma cells [61]. Likewise, the plant polyphenol downregulated expression of MMP-2, integrin receptors, focal adhesion kinase (FAK), and MT1-MMP to almost background levels in laryngeal squamous carcinoma cells, leading to significantly reduced invasive potential of the tumor cells. After drug withdrawal, expression of MMP-2, integrin receptors, MT1-MMP, and FAK, an important component of the intracellular signaling pathway, returned to control levels [55]. In human colon cancer cells, Curcumin diminished MMP-2 expression and promoted MMP-9 expression, but did not affect MMP-7, based on Western blotting assays on protein levels and confirmed by cDNA microarray on the respective mRNA levels [56]. Curcumin-treated prostate cancer cells (DU-145) had significantly reduced MMP-2 and MMP-9, along with impaired in vitro cellular invasion. In a corresponding xenograft model, tumorigenicity was diminished upon Curcumin treatment [57]. The highly metastatic murine melanoma cells B16F10 exhibited significantly reduced MMP-2 activity after treatment with Curcumin for 15 days [58]. Expression of MT1-MMP and FAK were also reduced to almost background levels. MMP-2, MT1-MMP, and FAK did not return to control levels even after 28 days of drug withdrawal. In the same in vitro model, Curcumin inhibited invasion along with migration and, on the other hand, enhanced apoptosis [62,63]. Curcumin inhibited the TPA-induced mRNA expression of MMP-1, -3, -9, and -14 in glioma cells [64] and suppressed various cell survival and cell proliferative genes, including Bcl-2, cyclin D1, IL-6, COX-2, and MMP-9 on head and neck squamous carcinoma cells [65]. In a xenograft model of prostatic cancer, Curcumin treatment lead to a significant reduction of MMP-2 and MMP-9 expression, and the inhibition of the invasive ability of the tumor cells in vitro. In this study, Curcumin reduced markedly the tumor volume, along with MMP-2 and MMP-9 activity in the tumor-bearing site, and the number of metastatic nodules in vivo in contrast to the untreated control group [57]. In the presence of estrogen, Curcumin inhibits expression of estrogen receptor (ER) in MCF-7 breast cancer cells, along with ER downstream genes, including pS2 and TGFβ. In the same study, Curcumin downregulated MMP-2 along with upregulation of TIMP-1 (tissue inhibitor of metalloproteinase) [66].

Anti-invasive effects of Curcumin have been reported also on lung cancer: migration and invasion of A549 cells were reduced in a time- and concentration-dependent manner by inhibition of MMP-2, -9, and VEGF [17]. Additionally, these effects could be detected not only in vitro but also in vivo on 801D cells. The underlying molecular mechanism involved the Rac-dependent pathway, which was inhibited by Curcumin, resulting in a reduction of MMP-2 and -9 [67].

In summary, Curcumin modulates the levels of almost all MMPs, with minor differences in the efficiency and some variety with respect to the MMPs affected. This may be caused by differences in responsiveness and partial resistance to Curcumin in some cell types, as we have shown previously in metastatic melanoma cells [23]. However, through the modulation of MMP/TIMP expression and activity, Curcumin considerably inhibits degradation of components of the basal membrane and the extracellular matrix, proving a rationale for reduced tumor invasion and growth.

### 2.3. Curcumin’s Anti-Inflammatory Action and Its Impact on Tumor Progression

Already in the late 19th century, Rudolf Virchow proposed the contribution of inflammation to tumor progression, which has been confirmed recently by new evidence [68]. Cancer and inflammation are connected by the intrinsic (activated oncogenes) and extrinsic (inflammation, infections) pathways leading to the activation of the three transcription factors: NFκB, STAT3 (signal transducers and activators of transcription 3), and HIF1α (hypoxia-induced factor 1α) [69]. There is substantial evidence that Curcumin modulates NFκB [70,71], and that most of the anti-inflammatory activities of the polyphenol can be attributed to the inhibition of the IκB kinase, IKK. Upon phosphorylation by IKK, IκB translocates into the proteasome, where it becomes degraded. In the absence of IκB, the NFκB subunit p65 becomes phosphorylated and translocates into the nucleus, where it can activate transcription of a large number of inflammation- and survival-related genes. It has been described that Curcumin inhibits STAT3 [72,73] and HIF1α directly, as well as indirectly through aryl hydrocarbon receptor nuclear translocator [74,75]. We have published previously that IL6—a downstream target of STAT3—is regulated by Curcumin [15].

Through reducing the activity of the transcription factors NFκB and STAT3, Curcumin determines a diminished expression of inflammatory cytokines [14,15], which in turn could decrease the attraction of inflammatory cells to the tumor site.

NFκB, TNFα, and its downstream target COX2 are known to be key players in inflammation, and, at the same time, they promote tumor growth and metastasis by triggering proliferation and angiogenesis and impede apoptosis [76]. We and others have found that Curcumin inhibits NFκB expression and activity, and decreases COX2 levels, both key events in inflammation, as well as cancer progression [15,16,31,70,77]. Inhibition of NFκB occurs through modulation of the IκBα pathway, which is directly targeted by Curcumin [78]. Through the suppression of NFκB, a variety of inflammatory cytokines, which have been shown to mediate tumori­genesis, are modulated by Curcumin. In addition, the natural polyphenol inhibits the expression of a series of interleukins (IL-1, -2, -5, -8, -12, -18) [79,80,81,82,83], which are significantly involved in the induction of MMPs, adhe­sion molecules, and signaling pathways related to invasion and an-giogenesis, like NFκB, TNF, and STATs.

**Figure 3:**
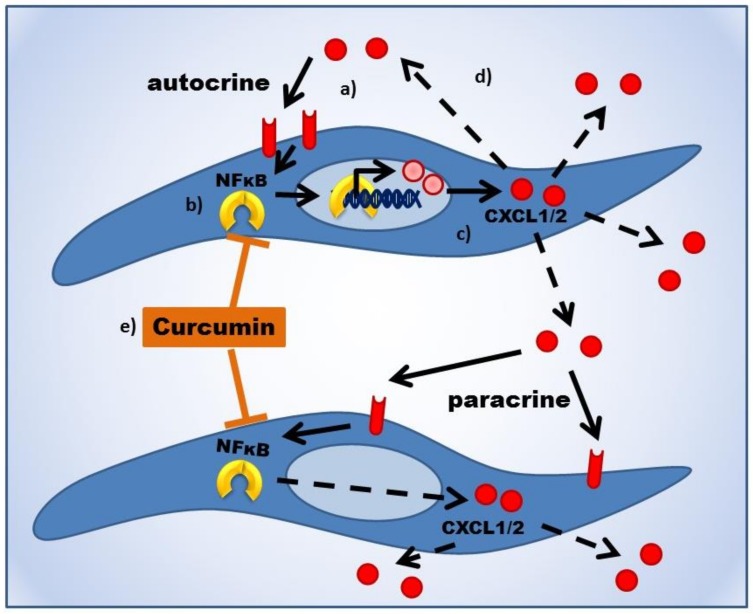
Feedback loop of CXCL-1 and 2 and NFκB. The proinflammatory chemokines CXCL-1 and -2 activate NFκB (**b**) by binding to their receptor CXCR-2 (**a**). In consequence, NFκB translocates into the nucleus and starts transcription of CXCL-1 and 2 (**c**). The chemokines are secreted and bind again to CXCR-2, activating NFκB (**d**). Curcumin disrupts this feedback loop by inhibition of NFκB (**e**).

By gene silencing of the NFκB subunit p65, we found that inhibition of CXCL-1 and -2 expression (CXCL-1: chemokine C-X-C motif ligand 1) by Curcumin in breast cancer cells is mediated through NFκB [15]. Furthermore, we showed that this mechanism requires intact IκBα expression upstream of NFκB. Well in line, we found that Curcumin inhibits translocation of NFκB to the nucleus through the inhibition of the IκB-kinase (IKKβ), leading to stabilization of the inhibitor of NFκB, IκBα, in PC-3 prostate carcinoma cells. Thereby, p65 is not translocated into the nucleus, whereby expression of CXCL-1 and 2 and the autocrine/paracrine loop that links the two chemokines to NFκB are abolished. The combined application of Curcumin with the synthetic IKKβ inhibitor SC-541 in prostate cancer cells did not show any additive or synergistic effects, indicating that the two compounds share the target (Figure 3) [14].

Inactivation of NFκB—which occurs in the tumor cell, as well as in the inflammatory cell—might be the reason why Curcumin, which is known to have only modest pharmacological effects, has these relatively strong chemopreventive properties, most likely synergistically interrupting the cross-stimulatory effect of cytokine production.

Interestingly, the downregulation of the cytokine CXCL-1 leads to reduced expression of CXCR-4, the receptor for CXCL-12/SDF1 [15], addressing a metastasis-promoting axis that has been recognized as a target for drug development [84].

Although CXCL-1 is expressed by only very few breast cancers [85], it is present in the lung metastasis signature [86]. Thereby, the cytokine might be a suitable response marker for secondary prevention by Curcumin in other cancer types that frequently overexpress CXCL-1.

### 2.4. Curcumin Modulates MicroRNA Expression

In ambition to identify novel target pathways to describe the antitumorigenic properties of Curcumin, the system of microRNAs cannot be neglected. MicroRNAs (miRNA or miR) are highly conserved, noncoding RNAs composed of 20–22 nucleotides, which are encoded in the genome of plants, animals, and humans.

It has been speculated that miRNAs serve as rivals to the commonly known system of transcription factors in eukaryotic cells, regulating the expression of about 60% of mammalian genes [87,88,89]. miRNAs are involved in numerous processes of development, differentiation, proliferation, and apoptosis, to mention only a few. Their involvement in several human diseases—in particular cancer [90,91], with some miRNAs being up- and others downregulated—has been described in literature [92,93,94]. They can regulate protein expression post-transcriptionally by controlling the expression of several proteins through binding to the 3′ UTR of the respective mRNA, thus resulting in mRNA degradation or inhibition of mRNA translation [95]. miRNAs can act as either tumor promoters (“oncomiRs”–miRNAs that act as oncogenes) or tumor suppressors (tumor suppressor miRs) [90,91,96]. In this context, miRNAs can modulate every single step of tumor progression, from proliferation and tumor cell growth to migration and invasion, and several miRNAs have even been associated to metastasis (“metastamirs”) with both prometastatic and antimetastatic effects [97,98].

One of the first preclinical studies to test the effect of Curcumin on miRNA expression has been performed by Sun and coworkers. In a microchip test for the most important—and potentially relevant—miRNAs, they found that Curcumin upregulated miRNA-22 and downregulated miRNA-199a in human pancreatic cancer cells (cell line PxBC-3) [99]. Accordingly, they suggested that Curcumin interferes with the miRNA system in human cancer cells, providing an additional anticancer effect yet to be explored in depth. In our own studies on metastatic breast cancer cells, we showed that Curcumin modulates the expression of miR181b, along with a series of other miRNAs. Investigating the consequences of miR181b modulation on tumor progression and metastases, we found that miR181b downmodulates CXCL-1 and -2 through a direct binding to their 3′-UTR. We could prove a direct correlation between the cytokines and miR181b by overexpression or inhibition of miR181b in metastatic breast cancer cells, which had a significant impact on CXCL-1 and -2 expressions and subsequently on metastasis formation in vivo in immunodeficient mice [21].

It has been shown that antiproliferative and proapoptotic effects of Curcumin are in part mediated by miRNAs. By upregulating miR192-5p, Curcumin suppresses the P13K/Akt signaling pathway and thereby inhibits proliferation and induces apoptosis in NSCLC cells [100]. Well in line, the tumor suppressor PTEN, a negative regulator of P13K/Akt, can be induced by Curcumin through suppressing miR-21 [101]. Other signaling pathways and factors that control proliferation and apoptosis comprise caspase-10, which is under the control of miR-186. By upregulating this miRNA, Curcumin inhibits progression of lung adenocarcinoma [102]. Downregulation of WT1 (Wilms tumor 1) by Curcumin through upregulation of miR-15a and miR-16-1 reduces proliferation in leukemic cells [103].

Besides acting on the P13K/Akt pathway and thereby on proliferation and apoptosis, Curcumin modulates also cell cycle arrest by promoting p21 via inhibition of miR-208 in prostate cancer cells [104]. Other miRNAs that are modulated by Curcumin and have impact on cell cycle, in particular on G2/M phase arrest, are miR21, an oncomiR, significantly increasing the number of viable cells, and miR-34a, which are both decreased by Curcumin [105,106].

As miRNAs are known to regulate invasion and metastases formation and Curcumin is known to inhibit these processes, it is more than likely that the anti-invasive and antimetastatic effects of the polyphenol are at least in part mediated by miRNAs.

In our own studies, we have demonstrated that miR181b inhibits expression of the matrix-degrading enzymes MMPs (matrix metalloproteinases), leading to reduced tumor cell invasion. We found that miR181b overexpression in metastatic breast cancer cells downregulates expression of genes belonging to the breast cancer lung metastases signature [86], and that miR181b overexpression in breast cancer cells inhibits metastases formation in vivo [21]. Similar findings were made on nasopharyngeal carcinoma (NPC), where Curcumin reduced formation of metastases by downregulating the expression of miR-125a-5p [107]. Interestingly, Curcumin analogs also have been found to inhibit invasion and metastases through miRNAs [108,109].

Altogether, these findings demonstrate that the antitumorigenic activity of Curcumin is mediated at least to some extent by miRNAs, which regulate a series of complex tumor progression-associated signaling pathways, like Akt, PTEN, Bcl-2, p53, Notch, and Erbb. Through a clear understanding of the relation between Curcumin and miRNAs, we can improve therapeutic strategies and find biomarkers for their efficacy. Thereby, the use of Curcumin will enrich the possibilities for more efficient and less toxic cancer therapies.

## 3. Metastatic Setting—A Possible Preventive Approach by Curcumin

In the last 15 years since Douglas Hanahan and Robert Weinberg described, for the first time, the hallmarks of cancer [110], various aspects of the metastatic program have been elucidated, particularly for carcinomas, which account for about 80% of cancer cases and the majority of cancer deaths. Cancer research can be regarded as an increasingly logical science, in which many phenotypic complexities result from a small set of underlying organizing principles [111]. Nevertheless, metastatic growth is still regarded as a multistep process, beginning with dissemination of carcinoma cells from the primary tumor site and ending with the arrival and growth in a distant tissue or organ.

Motility, degradation of basement membrane and extracellular matrix components, and invading into blood or lymphatic vessels are prerequisites for a tumor cell to metastasize.

During this process, many steps occur, like degradation processes, invasion, interactions with other cells, and many more.

Data from various tumor models underline the antimetastatic effect of Curcumin. First experiments using Curcumin as an antimetastatic agent were made by Menon and coworkers in 1995 [112], who tested in an in vivo model to see if Curcumin was able to inhibit the formation of lung metastases by B16F10 melanoma cells. In this study, Curcumin, orally administered in combination with catechin at “physiological” concentrations, was found to inhibit the formation of lung metastases by 80% and to increase the lifespan of animals by approximately 150%. In addition, Curcumin proved to be the strongest drug out of a dozen substances. In an orthotopic mouse model, implanted metastatic Lewis lung carcinoma (LLC-MLN) cells, selected in vivo, resulted in greater metastatic growth in mediastinal lymph nodes as compared to the original population [113]. By oral administration of Curcumin, mediastinal lymph node metastasis of implanted LLC cells was significantly inhibited without affecting the tumor growth at the implantation site. Even better results were obtained when Curcumin was administered in combination with the anticancer drug cis-diamine-dichloroplatinum (CDDP), which markedly inhibited tumor growth at the implanted site and lymphatic metastasis, and significantly prolonged survival.

The effect of Curcumin on DNA damage and lipid peroxidation was tested in vivo in a rat model of liver tumor resulting from copper-induced oxidative stress [114]. In this study, Curcumin suppressed formation of metastases, although it did not influence the tumor incidence of the primary liver tumors. Similarly, upon Curcumin treatment, significantly fewer metastases in a tumor model of prostatic carcinoma cells (DU-145) injected into immunodeficient mice [57] were observed, and in paclitaxel (Taxol)-resistant breast cancer cells a significant reduction in the incidence of breast cancer metastasis was reported.

Our own studies [14,15,16,21] revealed that formation of breast and prostate cancer metastasis in vivo was significantly reduced (Mann Whitney *p* < 0.01) in immunodeficient mice 5 weeks after injection. Surprisingly, four Curcumin-treated and none of the control animals in the breast cancer model remained metastases free.

In order to investigate the underlying molecular mechanisms, we analyzed all genes differentially expressed between Curcumin-treated and untreated MDA-MB-231 breast cancer cells. Our own microarray mRNA expression experiments resulted in 62 genes that were statistically significantly regulated by Curcumin, with the two proinflammatory cytokines, CXCL-1 and -2, among the strongest downregulated genes [15]. Both play a significant role in migration, growth, metastasis, and angiogenesis of tumor cells of different organs [115,116]. Our results were well in line with those previously published showing highly metastatic subclones derived from MDA-MB-231 cells by dilution cloning overexpress CXCL-1 and -2. However, the question of whether expression of these chemokines is causally linked to invasion and me­tastasis remained open [86]. Therefore, we validated our previous findings in a model of metastatic prostate cancer and investigated whether CXCL-1 and -2 are causally correlated with formation of metastases. Treatment of the cells with Curcumin and siRNA-based knockdown of CXCL-1 and -2 induced apoptosis, inhibited proliferation, and downregulated several important metastasis-promoting factors, like COX2, secreted protein acidic and rich in cysteine (SPARC), and EGF-containing fibulin-like extracellular matrix protein (EFEMP). In an orthotopic mouse model of hematogenous metastasis, treatment with Curcumin statistically significantly inhibited formation of lung metastases. Including the involvement of small noncoding RNAs (miRNAs) in tumor progression in our further studies concerning the molecular mechanisms of metastases formation and prevention by Curcumin, we demonstrated that the natural polyphenol modulates the expression of miR181b, along with a series of other miRNAs in metastatic breast cancer cells. Investigating the consequences of miR181b modulation on tumor progression and metastases, we found that miR181b downmodulates CXCL-1 and -2 through a direct binding to their 3′-UTR. We could prove a direct correlation between the cytokines and miR181b by overexpression or inhibition of miR181b in metastatic breast cancer cells, which had a significant impact on CXCL-1 and -2 expressions and subsequently on metastasis formation in vivo in immunodeficient mice [21].

We have developed the following model for the chemopreventive effect of Curcumin against formation of metastases (Figure 1 and Figure 2) based of our own results. By targeting its upstream inhibitor, IκBα, Curcumin acts on the tumor progression- and inflammation-related NFκB pathway. Inhibition of the transcription factor NFκB—along with other transcription factors, e.g., AP-1—leads to diminished expression of matrix-degrading proteases, like MMPs, and in consequence to reduced invasive capacity of the tumor cells. Additionally, Curcumin induces apoptosis by reducing the expression of NFκB-dependent survival factors. Through NFκB, Curcumin also impairs the synthesis of the proinflammatory cytokines CXCL-1 and -2, and, downstream that, of a series of metastasis-related genes. The concerted action of all these molecular processes leads to diminished formation of metastases in vivo.

## 4. Perspective

Curcumin has been used as dietary spice for centuries, even for more than 5000 years, in ayurvedic medicine and traditional Chinese medicine to treat inflammatory diseases. Its potential in cancer prevention and therapy is supported by numerous preclinical studies, and even in some clinical studies or healing attempts. However, there are few studies that clearly indicate adverse effects in cancer treatment. Therefore, future studies should be directed not only towards efficacy but also towards safety of Curcumin in preventing tumor progression and metastases formation.

As the prevention triad comprises not only primary prevention—thus intervention/action before the onset of cancer—but also secondary and tertiary prevention, we should start to pave the way for the application of Curcumin in the clinical setting.

At present, the major obstacles of using Curcumin in cancer prevention and treatment trials are its low bioavailability due to its hydrophobic character and its rapid metabolic rate [117]. Although new derivates and nanoparticles of Curcumin—like liposomes, polymeric micelles, and nanoparticles—have been designed in the last decade, the use is still restricted due to sometimes severe side effects of these new formulations.

Curcumin has demonstrated considerable antitumor effects in various tumor cell models. There is remarkable concordance between preclinical in vitro/in vivo studies and first clinical phase II trials in end-stage cancer patients, and it appears that Curcumin indeed exerts, at least in some tumors, clinically relevant anticancer effects. With ongoing research in the future, the list of tumor types that respond to Curcumin is likely to grow further.

Our own results reveal that Curcumin acts on apoptosis, expression of matrix-degrading enzymes, invasion, and expression of inflammation-related genes. The concerted action of a variety of molecular processes converges into diminished formation of breast and prostate cancer metastasis, as we have shown for the first time in a mouse model of hematogenous metastasis.

Formation of metastasis and metastatic lesions is the final step of a largely incurable illness, and the possibility to prevent this situation is at present the only chance for patients to prolong lifespan and quality of life. At present, we understand metastatic disease as a multistep process predominantly driven by dissemination, invasion, and colonialization.

The ideal chemopreventive agent against metastatic disease should therefore act against these three processes and not exert adverse effects. This review has extensively shown that Curcumin is effective against invasion and growth of tumor cells. Thereby, Curcumin has the potential to modulate the processes of invasion and colonialization. Dissemination of carcinoma cells is strongly influenced by interactions during transit through the circulating system. In this context, the escape from the arms of the immune system is essential for the tumor cell to be able to settle at a distant location for colonialization. Curcumin has been shown to exert immunomodulatory effects on several cells and organs of the immune system and is therefore recognized as a potent modulator of the immune system [118]. Additionally, we have summarized in this review irrevocable evidence that Curcumin is a potent inhibitor of metastases formation.

Due to the overwhelming evidence from preclinical studies, we think that the next step is to test Curcumin in clinical trials in order to prove its antitumor activities. However, some aspects have to be considered to elevate the success rate or to prevent failure, like in the case of the SELECT (selenium and Vitamin E cancer prevention trial) study, where vitamin E and selenium were tested as chemopreventive agents against prostate cancer [119,120,121]. With all the knowledge we have gained from preclinical studies on the molecular mechanisms of Curcumin’s antitumor activities, trials should be limited to cancer types with a documented role of NFκB and inflammation in tumor progression. In order to translate the preclinical finding to clinical evidence, the primary cancer should be available for molecular analysis and expression studies. In order to gain better insight into the bioavailability, Curcumin and its metabolites should be monitored in human specimens. Finally, patients should be stratified based on molecular and biochemical data in order to detect significant responses in subgroups of patients.

The still incompletely resolved issue of bioavailability should be addressed in future preclinical and clinical studies, since we need to attribute Curcumin’s antitumor activities to specific molecules circulating in the blood or present in the target organs. Another issue that has to be addressed in future studies is resistance against Curcumin, which can be expected more frequently when administered to patients for longer time periods, including innate resistance in some tumors that are known as difficult to treat, such as melanoma, as we have published previously [23].

Attention should also be paid to combinations of Curcumin with chemotherapy [122,123] or with other plant-derived bioactive substances with preclinically proven antitumor activities. In this context, clinical studies should be carried out to collect scientific evidence for clinical application also with respect to Curcumin’s protective features against undesired side effects [124,125,126,127,128] or its reinforcing power in combination with classic antineoplastic therapies [129].

Besides still missing evidence from large clinical trials, Curcumin, like other natural plant-derived bioactive compounds, has further two major disadvantages that render it commercially unattractive: it is too cheap and nonpatentable. However, the comprehensive data from preclinical studies, together with first clinical results from single patients or small cohorts, makes it very likely that Curcumin can contribute significantly to the improvement of cancer therapy and prevention.
